# Supplementary material for: Correlation of Infinium HumanMethylation450K and MethylationEPIC BeadChip arrays in cartilage
Source: Epigenetics. 2019 Dec 13;15(6-7):594–603. doi: 10.1080/15592294.2019.1700003 (PMC7574380; doi:10.1080/15592294.2019.1700003)
Supplement: Supplemental Material [file KEPI_A_1700003_SM2115.zip › Suppl files.docx]

**Supplemental Figure 1.** **Comparison of methylation levels in 21 cartilage samples measured by 450K array, EPIC array, and pyrosequencing for six CpG sites**. (A) and (B) Methylation levels (A) cg24555089 and (B) cg20806296, both of which have very different β values for the same 21 samples between the 450K and EPIC arrays. Left graph, 450K vs EPIC β value; middle graph, 450K vs pyrosequencing % methylation; right graph, EPIC vs pyrosequencing % methylation. The dotted green line represents when the methylation levels detected by the two techniques are the same. The accuracy of each pyrosequencing assay was validated using a calibration curve of known methylation values of 0-100%. (C) Table of the mean methylation levels measured by the two arrays and pyrosequencing for all six CpGs assayed. β value is given for the two arrays, and the percentage methylation measured by pyrosequencing has been converted to a β value. The Root Mean Square Error (RMSE) value is given for the pairwise comparison between two methods; the smaller the value, the more similar the β values quantified by the two methods are. DNA methylation levels were corrected if the calibration curve deviated from the expected value, and these corrected values were used to determine the RMSE, except for cg 13782176 for which the uncorrected methylation levels were used.

**Supplemental Figure 2.** Comparison of methylation levels in 21 cartilage samples measured by 450K array, EPIC array, and pyrosequencing for (A) cg19254793, (B) cg1397908, (C) cg20913747 and (D) cg13782176. Left graph, 450K vs EPIC β value; middle graph, 450K vs pyrosequencing % methylation; right graph, EPIC vs pyrosequencing % methylation. The dotted green line represents when the methylation levels detected by the two techniques are the same. The accuracy of each pyrosequencing assay was validated using a calibration curve of known methylation values of 0-100%. The calibration curve for cg1925474, cg13979708, and cg13782176 deviated from the expected value, and therefore the measured pyrosequencing results of the samples were corrected. Both the (i) uncorrected and (ii) corrected pyrosequencing methylation levels for cg13782176 are shown in (D).

**Supplemental Table 1**. **Correlation of CpG sites across 450K and EPIC arrays in three tissue types.** Common samples assayed on both 450K and EPIC arrays were investigated in whole blood (GEO GSE86833) and pediatric brain tumour (GEO GSE92580). (A) Percentage of total numbers of CpG probes grouped by Pearson’s *r***.** (B) Mean and median Pearson *r* values per tissue for (i) CpGs with *r* >0.8 and (ii) *r* <0.2 in at least one tissue. (C) The grouped distribution of Pearson’s *r* values in cartilage, tumour and whole blood of low correlating probes (r ≤0.2) identified in 108 placental samples by Fernandez-Jimenez and colleagues (ref). (D) Number of differentially methylated CpGs identified between NOF and OA knee cartilage using different Δβ thresholds. For overlapping CpGs identified by both 450K and EPIC arrays, the mean and median Pearson r is shown.
