# Supplementary material for: Correlation of Infinium HumanMethylation450K and MethylationEPIC BeadChip arrays in cartilage
Source: Epigenetics. 2019 Dec 13;15(6-7):594–603. doi: 10.1080/15592294.2019.1700003 (PMC7574380; doi:10.1080/15592294.2019.1700003)
Supplement: Supplemental Material [file KEPI_A_1700003_SM2115.zip › Suppl files.pptx]

## Slide 1
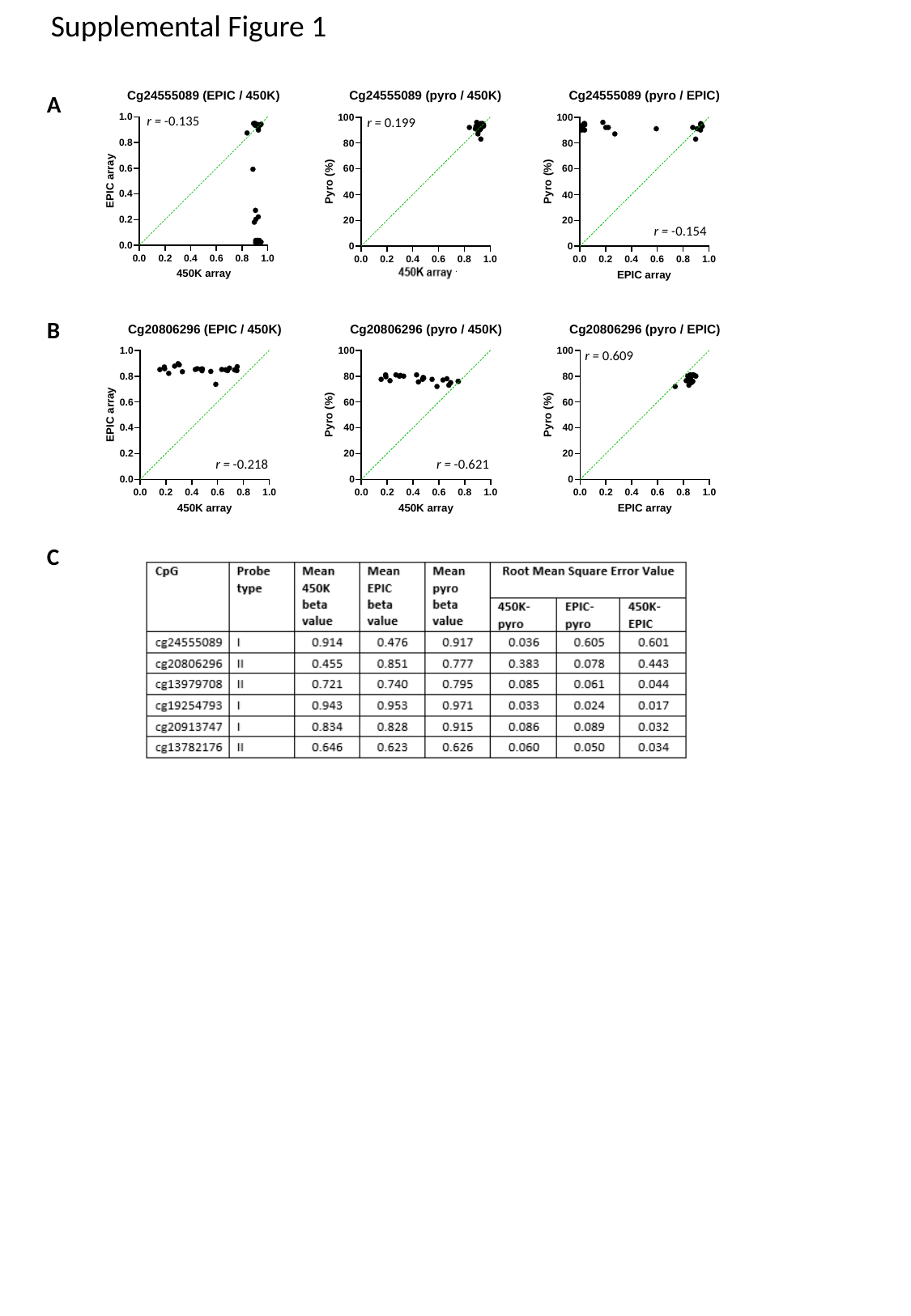

Supplemental Figure 1
A
B
C
r = -0.135
r = 0.199
r = -0.154
r = 0.609
r = -0.218
r = -0.621

## Slide 2
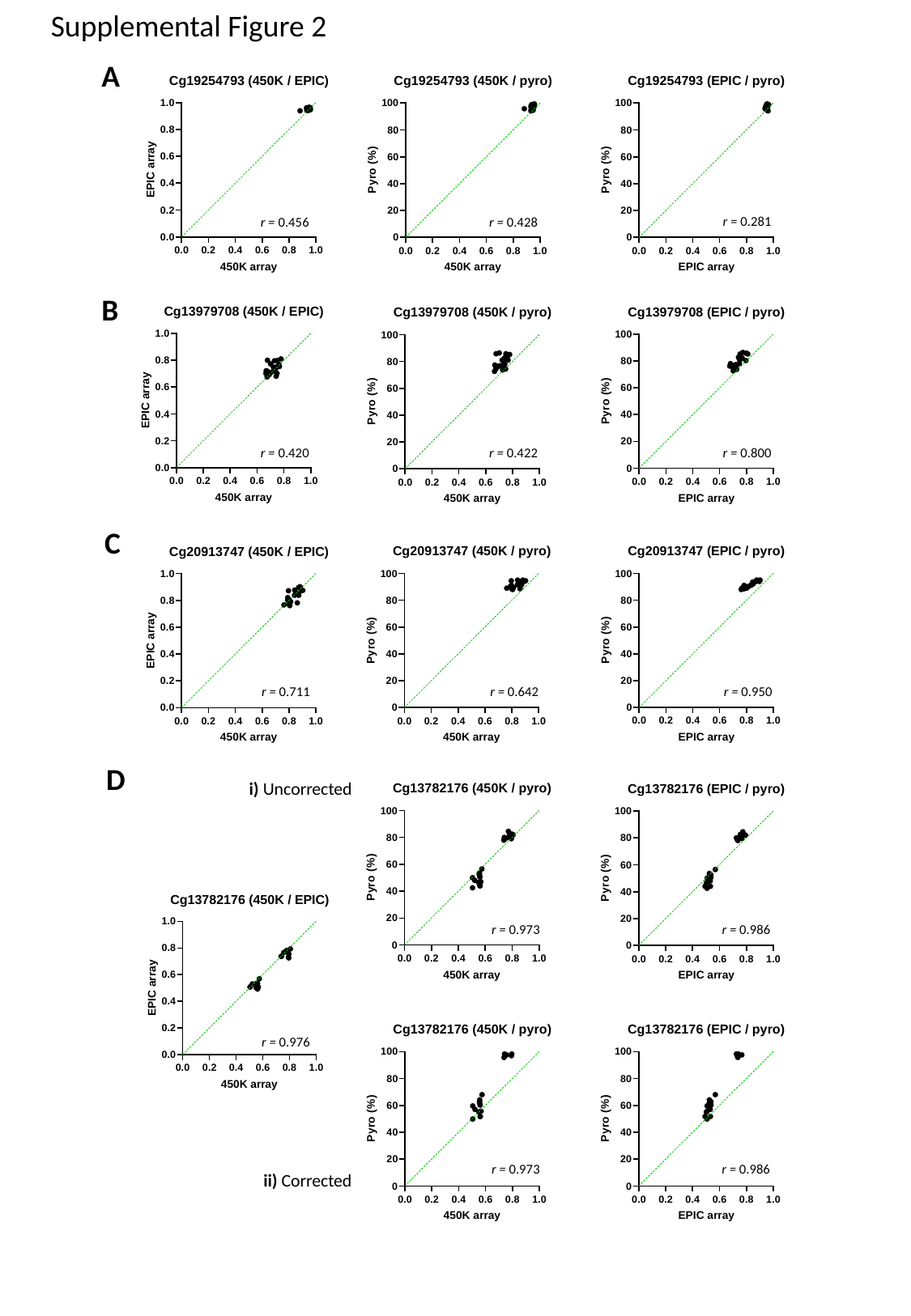

Supplemental Figure 2
A
B
C
D
i) Uncorrected
ii) Corrected
r = 0.281
r = 0.456
r = 0.428
r = 0.800
r = 0.420
r = 0.422
r = 0.950
r = 0.711
r = 0.642
r = 0.973
r = 0.986
r = 0.976
r = 0.973
r = 0.986

## Slide 3
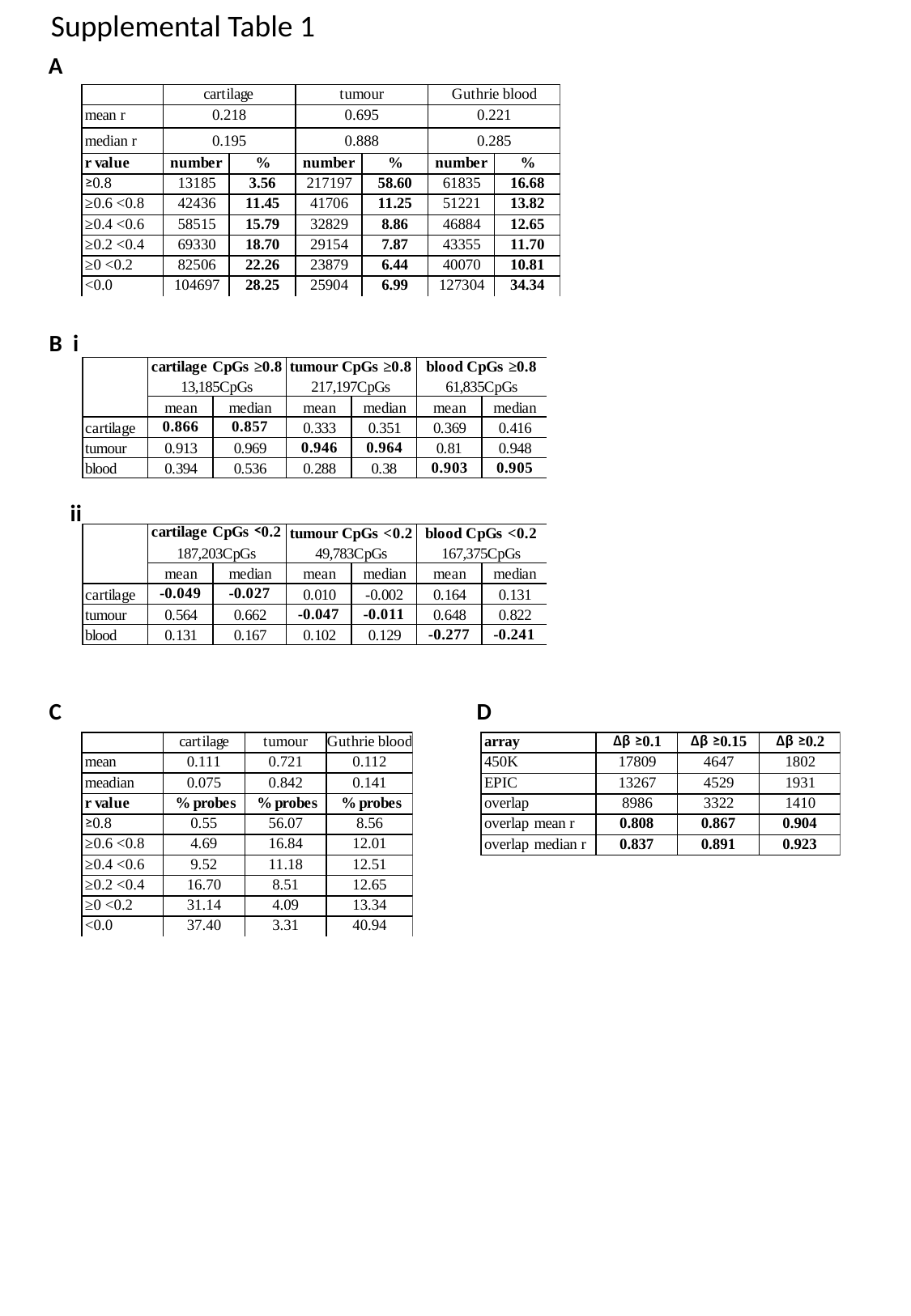

Supplemental Table 1
A
B i
 ii
C D

## Slide 4
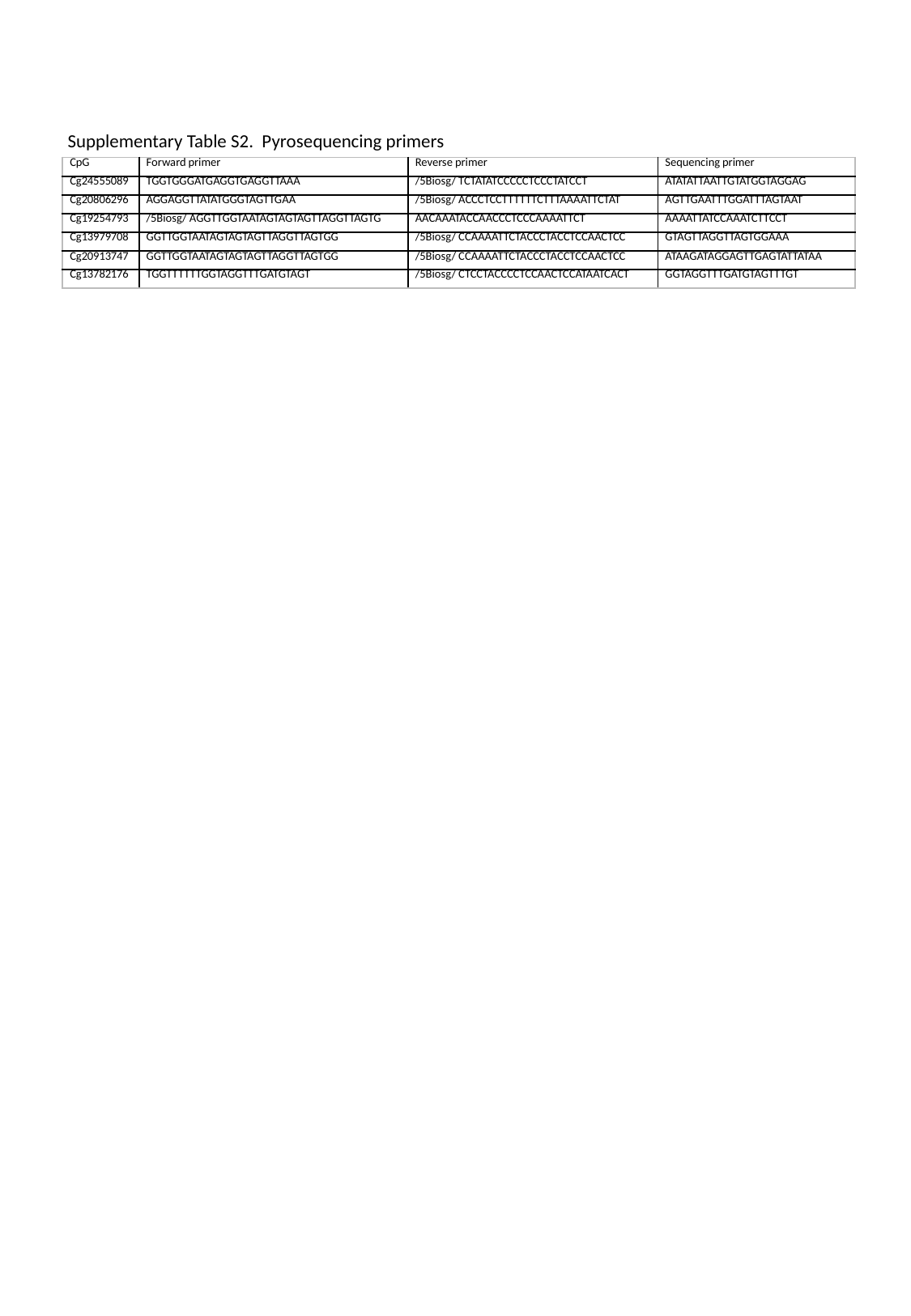

Supplementary Table S2. Pyrosequencing primers
| CpG | Forward primer | Reverse primer | Sequencing primer |
| --- | --- | --- | --- |
| Cg24555089 | TGGTGGGATGAGGTGAGGTTAAA | /5Biosg/ TCTATATCCCCCTCCCTATCCT | ATATATTAATTGTATGGTAGGAG |
| Cg20806296 | AGGAGGTTATATGGGTAGTTGAA | /5Biosg/ ACCCTCCTTTTTTCTTTAAAATTCTAT | AGTTGAATTTGGATTTAGTAAT |
| Cg19254793 | /5Biosg/ AGGTTGGTAATAGTAGTAGTTAGGTTAGTG | AACAAATACCAACCCTCCCAAAATTCT | AAAATTATCCAAATCTTCCT |
| Cg13979708 | GGTTGGTAATAGTAGTAGTTAGGTTAGTGG | /5Biosg/ CCAAAATTCTACCCTACCTCCAACTCC | GTAGTTAGGTTAGTGGAAA |
| Cg20913747 | GGTTGGTAATAGTAGTAGTTAGGTTAGTGG | /5Biosg/ CCAAAATTCTACCCTACCTCCAACTCC | ATAAGATAGGAGTTGAGTATTATAA |
| Cg13782176 | TGGTTTTTTGGTAGGTTTGATGTAGT | /5Biosg/ CTCCTACCCCTCCAACTCCATAATCACT | GGTAGGTTTGATGTAGTTTGT |
